# Indents

**Published:** 1916-02

**Authors:** 


### INDENTS

☞ **Brief Articles of Technical or Scientific Value will receive notice and comment. Contributions invited.**

*Stypticin Dental Tablets.* These tablets are prepared in suitable form for dental purposes by Merck & Co., of New York. Each tablet contains three-fourths of a grain.

*Arsenic in Proximal Cavities.* Place a piece of temporary gutta percha stopping against the gum in the inter-proximal space and force it tight between the teeth and below the cavity margins. Then apply the nerve paste and cover with cement.—A. C. Sloan in *Digest.*

*Root Canal Filling.* Dissolve one half ounce of gutta percha baseplate in chloroform, make a one-half ounce saturated solution of thymol in Eucalyptol and add it to the chloropercha and mix thoroughly. Allow the chloroform to evaporate. Dry the root canal thoroughly and work the paste into the canal with a warm broach, forcing it to the apex with a bit of soft rubber. Then force into the canal a gutta percha canal point and seal with desired filling.—Dr. J. C. Hopkins in *Pacific Gazette.*

*The Joy of Intelligence.* One thing that has been a great help to me in my practice is that a thorough knowledge of any subject which has found definite lodgment in my consciousness is an asset that is far more valuable to me than all shiftless and careless methods that may be employed for the purpose of doing work quickly; an asset of more value than a finely equipped office, a distinguished appearance, or any advertising. To be able to explain intelligently the cause of any trouble, and outline definitely a method of reconstruction and repair, and to be able to give a rational reason for my methods, is most convincing and is conducive of fees that justify the time spent in working out the details of such restorations.—R. J. Reinhart in *Western Journal.*

*Sanitation in the Schools.* By a recent decision of the children's court in Brooklyn, N. Y., the first of its kind in the state, the authority of the board of education to compel parents to send their children to school in as good physical condition as possible is sustained.

The parents of a pupil were ordered by the court to have the boy's diseased tonsils removed. They had ignored frequent complaints from teachers that the boy was incapable of making progress in his education unless his tonsils were treated.

This is good news, and hereafter no parent will have the right to expose other pupils to a possible contagion or humiliate a child who is incapable of making progress in his school work because of physical defects that can be remedied. This will apply to dental defects as well, and the board will have authority to compel parents to remedy such when they retard the child's progress in school work and a menace to other children with their uncared-for mouths. This decision is an important one and likely to be far-reaching in its effect.—*Oral Hygiene.*

*Why We Prematurely Break Down.* If you are nearing or past forty, and are wise, you will consult your physician once a year, submit yourself to a thorough examination (including that of the urine) and carefully follow the advice he gives you. Don't wait until you notice suspicious symptoms; that is usually too late.

In conclusion let me warn you of the dangers of overeating. Most of us eat too much. We would do well to follow the advice of the great English physician, George Cheyne: *"Every wise man,* after *fifty,* ought to begin to lessen at least the quantity of his aliment, and if he would continue free of great and dangerous distempers and preserve his senses and faculties clear to the last, he ought every seven years to go on abating gradually and sensibly, and at last descend out of life as he ascended into it, even into the child's diet." In short, why do men over forty break down? Indulging their appetites!—Dr. C. F. Boldwan.

*Amalgam Fillings.* Most of us have in the past thought that undercuts of any sort were good enough for amalgam fillings. We probably considered amalgam a cheap material, and in consequence felt that its insertion did not justify a great amount of labor; but I desire to say that I have learned that amalgam fillings are not to be considered cheap, in the sense that they can not be made an efficient factor in tooth restoration. They may be less expensive than gold, but the preparation of the cavity for their reception is just as important as for gold, and time and skill are required for such proper insertion.

A systematic, carefully outlined cavity, filled with well condensed amalgam, tooth form carefully restored by bringing out contact point, marginal ridge, cusps and sulci and filling polished, is a piece of work which gives service, establishes satisfied patients, enriches and elevates our thought and produces a fee that enables us to be more than mere "hole fillers." Why can we not all co-operate in such a work and educate our patients so that they will appreciate the value of such services and thus be known as real benefactors? True, we can not do this work for one dollar a filling, or two dollars a filling, even in small towns or cities, but times are changing and we must all awaken from this stupidity of fear that cripples our capacities and enfeebles our forward steps and would keep us in the narrow path of poverty.—R. J. Reinhart in *Western Journal.*

*Shortage of Dental Practitioners in England.* The institution of a diploma in dental surgery took place comparatively recently in this country. The result is that the number of qualified dentists is far short of the requirements of the population, and that a large number of unqualified men practice. They are not prevented from doing this, but they must not call themselves dentists. This is a very small drawback, as they can exhibit sets of teeth and call themselves "tooth specialists." At a meeting of the General Medical Council, Mr. Tomes, chairman of the Dental Education and Examination Committee, submitted a report on the shortage of dentists. Communications had been made with the various licensing bodies for the possibility or curtailment of the curriculum without lowering the standard of dental practice. Some of the bodies questioned the existence of any shortage, pointing out that many qualified men are not fully occupied, the public being unconvinced of their advantage over the unqualified. Attention was also drawn to the lowering of the social status which arose from the intrusion of great numbers of unqualified persons, and to the fact that business men who had acquainted themselves with the existing state of things considered that from a business point of view qualification was worthless or even a hindrance, and so did not put their sons at dental schools. The Incorporated Dental Hospital of Ireland alone considered the possibility that the simpler dental requirements sought by the poorer classes might perhaps be met by a lower grade of practitioner, though this was also suggested in one of several letters sent by private practitioners. The main conclusion was that no appreciable increase in the members of the dental profession can be looked for until the law gives further protection to the qualified man against the unqualified. A very insidious form of deception was pointed out. An unqualified man dare not put on his plate "dental surgeon," as this would render him liable to prosecution. This is avoided by putting beneath his name "dental surgery," which can be done with impunity.—London Letter in *Journal A. M. A.*

*Confection for Administering Medicines.* I have experimented considerably to find a candy form that would lend itself to extemporaneous preparation, and yet yield tolerably stable products. I have found it in sugar tablets, for which I have proposed the name "sweet tablets," (Latin, tabellae dulces).

To prepare these, the pharmacist needs no greater equipment than a ten-dollar tablet machine. Where a universal formula for a base for sweet tablets is desired, it may be found in the following formula:

VANILLA CACAO SUGAR.

| | |
|---|---|
| Tincture of vanilla ........................ | 5.00 c.c. |
| Cacao powder ............................. | 10.00 gm. |
| Dextrose .................................. | 10.00 gm. |
| Sugar, powdered ........................ | 80.00 gm. |

Mix thoroughly by trituration in a mortar, and preserve in a well-stoppered bottle.

Mixing this sugar preparation with the proper amount of medicament in such proportion that it is contained in 0.30 gm. yields a powder that is ready for compression in the tablet machine.

For substances that have a slight flavor of their own, such as thyroid, sulphur or podophyllum, cinnamon cacao sugar is better. The latter may be prepared by substituting 0.5 c.c. of 10 per cent. spirit of cinnamon for the tincture of vanilla.

Inasmuch as variety in flavor and appearance is quite desirable in this form of medication, I have experimented to find a substitute for cacao that would lend itself as well to extemporaneous tablet making as that substance, when added to sugar. I have found it in what I propose to call "fat starch," for which the following formula might be given:

FAT STARCH.

| | |
|---|---|
| Alcoholic solution of saccharin, 3 per cent...... | 15.00 c.c. |
| Liquid petrolatum ........................ | 25.00 c.c. |
| Starch. ................................. | 75.00 gm. |

Mix the starch with the solution of saccharin, and permit the alcohol to evaporate completely. Then incorporate the liquid petrolatum.

This fat starch added to sugar to the extent of 20 per cent. yields a powder that is ready for compression in the tablet machine. By appropriate coloring and flavoring, quite a variety of sweet tablets can be elaborated.—Bernard Fantus M.D. in *J. A. M. A.*, Jan. 1, 1916.

*Commercializing the Dental Profession.* The following editorial from the *Bulletin* of the Indiana Dental Association is timely and should appeal to every loyal member of the dental profession. The evil cited is rampant in every dental society in the land and should be eradicated. Men who should be above such things are continually orating and demonstrating methods and processes they are careful to state can only be accomplished by the use of special instruments and materials which can only be obtained at stated places. Many of these enthusiasts are actually paid salaries for this form of professional prostitution:

"The dental profession, not only in Indiana, but everywhere, is becoming permeated with commercialism. If we continue to permit men to read papers and give clinics who have a 'nigger in the wood pile' proposition back of it, we will in the very near future lower our standard of ethics to that of a trade union.

"The time has come when men should be invited to appear on our programs on their record, as efficient men or scientific thinkers in the field of dentistry. We should carefully consider inviting any man that has a financial proposition back of his paper or clinic. It was disgusting to see the large number of men who gave clinics at the Panama-Pacific Dental Congress that had something to sell. In fact, there seemed to be a mercenary spirit in the background of nearly everything presented.

"If our officers are not acquainted with the men whom they desire to invite on the program, they should first consult others, until they are convinced that such men are not only capable, but have enough of the self-sacrificing spirit for the good of humanity, to be willing to give something to their brothers in dentistry, without the thought of being compensated for their services.

"The name of G. V. Black will ever live in the hearts of members of the dental profession as a man who gave his time and life, not for the sordid thought of sometime being compensated for his work, but in order that he might be of some service in uplifting humanity. Such a self-sacrificing life should be the guiding star of every dentist who has the good of his profession at heart. Upon such fundamental basic principles should our profession be builded and maintained. To the essayist or clinician who assists in maintaining such an ethical standard, the profession will manifest its spirit of appreciation. And such a one will be conscious of having discharged his duty to the profession and to the race.

"Since our profession is almost saturated with this materialistic spirit, it will mean that some officer must have the courage and determination to see that our next meeting is a strictly ethical meeting from every standpoint. If some of the other State societies desire to lower the standard of their profession, that may be permissible, but for a State organization such as Indiana has, it should stand for the highest ethical requirements.

"That dentist who has a financial 'axe to grind' at the expense of the ethics of his profession, should be treated as a parasite with the germicide of a higher ethical professional standard.

"Let's banish this form of commercialism and perfidious salesmanship from the dental profession of Indiana."—*Western Dental Journal.*

*Deaths of Physicians in 1915.* During 1915 the deaths of 2,451 physicians in the United States and Canada were noted in *The Journal.* Reckoning on a conservative estimate of 156,000 physicians, this is equivalent to an annual death rate of 15.71 per thousand. For the thirteen previous years the death rates were as follows: 1914, 14.41; 1913, 14.64; 1912, 14.13; 1911, 15.32; 1910, 16.96; 1909, 16.26; 1908, 17.39; 1907, 16.01; 1906, 17.20; 1905, 16.36; 1904, 17.14; 1903, 13.73 and 1902, 14.74. The average annual mortality for the period from 1902 to 1915 inclusive was, therefore, 15.71 per thousand. The chief death causes in the order named were the same as for last year, namely; senility, heart disease, cerebral hemorrhage, pneumonia, accident and nephritis. The age at death varied from 21 to 102, with an average of a little over 59 years. The general average of age at death since 1904 is 59 years, 6 months and 11 days. The number of years of practice varied from 1 to 79, the average being 32 years, 4 months and 26 days. The average for the past twelve years is 32 years, 2 months and 13 days.

*Causes of Death.*—There were 227 deaths assigned to general diseases; 276 to diseases of the nervous system; 293 to diseases of the circulatory system; 173 to diseases of the respiratory system; 115 to diseases of the digestive system; 124 to diseases of the genito-urinary system; 400 to senility; 44 to suicide; 104 to accident; 21 to homicide, and 88 after surgical operation. Among the principal assigned causes of death are senility, 400; heart disease and cerebral hemorrhage, each 218; pneumonia, 154; accident, 104; nephritis, 90; malignant disease, 65; tuberculosis, 62; suicide, 44; angina pectoris, 28; septicemia, diabetes, appendicitis and arteriosclerosis, each 22; homicide, 21; typhoid fever and gastritis, each 15; uremia, 14; meningitis and intestinal obstruction, each 12; myocardities, 11; cholecystitis, 10; influenza and paresis, each 8; anemia and bronchitis, each 6; rheumatism, insanity, endocarditis, peritonitis and prostatis, each 5; embolism, 4 typhus fever, dysentery, erysipelas, epilepsy, acute dilatation of the heart, gastric ulcer, hernia and cirrhosis of the liver, each 3; pellagra, leukemia, arthritis deformans, drug addiction, parlysis, paralytic dementia, mastoiditis, asthma, atrophy of the liver, ptomain poisoning and dropsy, each 2, and malaria, scarlet fever, diphtheria, tetanus, alcoholism, cerebro spinal fever, locomotor ataxia, neuritis, aneurysm, pleurisy and gangrene of the lung, each 1. Two physicians died in prison, and 9 lost their lives either in battle on land or by the sinking of ships on which they were medical officers.

The causes assigned for the 104 deaths from accident were: Automobile, 23; falls and drowning, each 16 (of the latter, 5 physicians were drowned in attempting to save lives); poison, 11; automobile and railway, each 10; burns, 4; firearms and by animals, each 2, and asphyxiation, sunstroke, privation, lightning and explosion, one each. The 44 physicians who ended their lives by suicide selected the following methods: Firearms, 17; poison, 15; cutting instruments, 5; cause not stated, 3; strangulation, 2, and asphyxiation and jumping from a high place, each one. Of the twenty-one homicides, 18 were due to firearms, one to hanging, and 2 to other means, and of these deaths, 7 occurred in feuds or affrays.

*Ages.*—Of the decedents, 69 were between the ages of 21 and 30; 220 between the ages of 31 and 40, 341 between 41 and 50; 485 between 51 and 60; 519 between 61 and 70;

415 between 71 and 80; 212 between 81 and 90; 30 between 91 and 100, while one physician attained the age of 102 years. The greatest mortality occurred at the age of 61, when 68 deaths were recorded; at 70 with 65 deaths; at 62 with 63 deaths; at 55 with 58 deaths; at 60 with 56 deaths; at 75 with 54 deaths; at 56, 58 and 64 with 53 deaths; at 54 with 51 deaths, and at 44, 59 and 66, when 50 died. There were 9 deaths at 91, 7 each at 92 and 93; 3 at 94; 2 each at 96 and 98, and one death at 102 years.

*Years of Practice.*—By periods of ten years, physicians died as follows during the year: In the first decade, 172, of whom 2 had been in practice less than one year and 16 less than two years; in the second decade, 356; third decade, 511; fourth decade, 598; fifth decade, 453; sixth decade, 221; seventh decade, 101, and eighth decade, 9, of whom 5 had been in practice 70 years, 2, 75 years, and one each 78 and 79 years.—*Journal A. M. A.*, Jan. 1, 1916.

